# Parent-reported health care expenditures associated with autism spectrum disorders in Heilongjiang province, China

**DOI:** 10.1186/1472-6963-12-7

**Published:** 2012-01-10

**Authors:** Jia Wang, Xue Zhou, Wei Xia, Cai-Hong Sun, Li-Jie Wu, Jian-Li Wang, Akemi Tomoda

**Affiliations:** 1 Department of Children's and Adolescent Health, Public Health College of Harbin Medical University, 157 Baojian Road, Nangang District, Harbin 150086, P.R. China; 2 Departments of Psychiatry and of Community Health Sciences, Faculty of Medicine, University of Calgary, Calgary T2N 4N1, Canada; 3 Department of Child Development, Faculty of Life Sciences, Kumamoto University, 1-1-1 Honjo, Kumamoto, 860-8566, Japan

**Keywords:** health care expenditure, autism spectrum disorders, family disease burden

## Abstract

**Background:**

The aim of this study was to determine the health expenses incurred by families with children with autism spectrum disorder (ASD) and those expenses' relation to total household income and expenditures.

**Methods:**

In this cross-sectional study, health care expenditure data were collected through face-to-face interviews. Expenses included annual costs for clinic visits, medication, behavioral therapy, transportation, and accommodations. Health care costs as a percentage of total household income and expenditures were also determined. The participants included 290 families with ASD children who were treated at the Children Development and Behavior Research Center, Harbin Medical University, China.

**Results:**

Families with ASD children from urban and rural areas had higher per-capita household expenditures by 60.8% and 74.7%, respectively, compared with provincial statistics for 2007. Behavioral therapy accounted for the largest proportion of health expenses (54.3%) for ASD children. In 19.9% of urban and 38.2% of rural families, health care costs exceeded the total annual household income. Most families (89.3% of urban families; 88.1% of rural families) in that province reported higher health care expenditures than the provincial household average.

**Conclusion:**

For families with ASD children, the economic burden of health care is substantially higher than the provincial average.

## 1. Background

Autism spectrum disorders (ASD) are a group of developmental disabilities characterized by pervasive deficits in socialization and communication, as well as unusual behaviors or interests [1]. Children with autism can present a broad range of clinical features including qualitative developmental and neurological abnormalities, as well as sensory/motor symptoms, epilepsy, cognitive dysfunction, severe impairments in adaptive behavior, and aberrant regulation of emotion [1,2]. ASD include autism, Asperger syndrome and pervasive developmental disorder not otherwise specified (PDD-NOS). The impairments range from mild to severe.

China has the world's largest population. Since four children with autism were first identified in Nanjing in 1982, interest in autism has increased. The number of newly diagnosed cases of ASD seeking medical services has increased substantially. Nevertheless, a lack of nationwide epidemiologic data on ASD persists in China. In some regional studies conducted in China, the prevalence of autism was 1.10 per 1000 children in the city of Tianjin (2004), 1.25 per 1000 children in Jiangsu province (2001), 1.34 per 1000 children in Beijing (2007) and 2.27 per 1000 in Harbin city (2010) [3-6]. It has since been estimated that there are about 7.8 million individuals with ASD in China, with more affected boys than girls [7].

Some medications including melatonin and risperidone have been used to treat ASD [8,9]. However, behavioral therapy is the most widely used therapeutic method for improving social and language skills in children with ASD [10]. Behavioral intervention plays an important role in ASD rehabilitation. With improved awareness and knowledge of ASD, Chinese parents of ASD children have recognized that it is important for their children to receive early intervention and behavioral therapy. Consequently, the number of parents seeking early intervention is increasing rapidly.

Public rehabilitation services in China are limited. Institutions involved in ASD behavioral therapy and training include medical and educational institutes and The China Disabled Person's Federation (CDPF). Private services are a supplementary component of the system. Most of the private services have been established and managed by the parents of ASD children.

The economic burden and the impacts on health and social integration associated with ASD are considerable. According to a UK study [11], the costs of supporting children with ASD were estimated as £2.7 billion per year. For adults, these costs amount to £25 billion per year. The lifetime cost was estimated as £1.23 million for individuals with ASD and intellectual disability and £0.80 million for individuals with ASD but without intellectual disability [11]. Similarly, in 2003, a US study estimated that the annual expenditure per autistic child for early medical service was $35,000 [12]. The US government has invested $3.2 million for children with autism to support expenses incurred for children aged 3-7 years old [12]. However, financial support from the government for children with ASD children is in a very early stage in China. The CDPF has planned to establish early rehabilitation and training settings for autistic children in 31 cities in China, but few developed cities provide financial support to the families involved. One obstacle is the lack of basic descriptive data about current costs of treatment of ASD in China and how these costs relate to total household income and expenditures.

To rectify this situation, we conducted the current study in Heilongjiang province, which is located in northeastern China. Its total area is 473,000 km^2^, with a population of 38.24 million, of which 46.1% reside in urban areas and 53.9% in rural areas. In terms of gross domestic product, Heilongjiang was ranked 15^th ^among 31 provinces in 2007. Generally, families in urban areas have higher income than families in rural areas in China. Therefore, it is possible that the per-capita health care expenditure and its share of household income differ between rural and urban areas. The provincial government is currently unable to provide sufficient financial support for rehabilitation therapy for all ASD children. In fact, the provincial government has limited economic assistance to families with low income. Nevertheless, most families with ASD children face significant economic stress. Accordingly, understanding the economic costs and burdens associated using services in this population will provide a strong basis for policy development.

In this study, we estimated and compared health care expenditures for physician visits and rehabilitation therapy incurred by families with ASD children in Heilongjiang province, overall and according to rural or urban areas. Data indicating health care costs as a percentage of household income and overall annual expense were collected and analyzed.

## 2. Methods

### 2.1. Study population

Parents whose children were diagnosed with and treated for ASD at the Children Development and Behavior Research Center (CDBRC), Harbin Medical University, were enrolled for this study. The CDBRC is a specialized and authorized institution that provides counseling, evaluation, training, rehabilitation, and health education for children with developmental disabilities and behavioral disorders. From May 2007 to August 2009, 290 children with ASD had received services provided by the CDBRC. Their parents were included in this study. ASD was diagnosed by pediatricians and psychiatrists according to the Diagnostic and Statistical Manual of Mental Disorders - IV diagnostic criteria [13]. This study was approved by the ethics review committee of Harbin Medical University.

### 2.2. Measures

All parents of ASD children who sought services provided by the CDBRC between May 2007 and August 2009 were invited to participate and completed a questionnaire (*n *= 290), which recorded the following information: per-capita household income in the past year (total household income divided by number of family members), per-capita household expenses, medical experience, behavioral therapy, and health care expenditures related to ASD treatment. Health care expenditures included costs of clinic counseling, diagnosis, behavioral intervention, medication, transportation, and accommodation. We also recorded demographic data for the children and the families, including sex, age, race, family structure, urban/rural areas, employment status, educational levels and developmental quotient (DQ).

The health care burden for the families with ASD children, we compared the household income and health care expenses of these families with provincial statistics for 2007. Data collected and published by the Statistics Bureau of the Central Government and are publicly available (http://www.stats.gov.cn/tjsj/ndsj/2008/indexch.htm).

### 2.3. Statistical analysis

Per-capita household income and expenditures and health service expenses were analyzed for the overall population and according to urban and rural areas. Specific health care expenses related to consultation, medication, behavioral therapy, transportation, and accommodation during the past 12 months were estimated and compared between families residing in rural and urban areas. Health care costs as a percentage of household income and overall annual expense were collected and analyzed. The percentages were also compared between rural and urban areas and with provincial statistics for 2007.

## 3. Results

The demographic characteristics of the children who received services at the CDBRC are presented in Table 1. During the study period, 290 children aged between 1.82 and 15.12 years received treatment at the CDBRC. The mean age was 5.44 (SD = 2.43) years old. There were more boys than girls. More families from urban areas participated than families from rural areas. The average DQ of the participants was 52.30 (SD = 16.25), ranging from 18 to 93. The mean age of participants' mothers was 32.10 (SD = 4.41) years old, ranging from 23 to 48 years old; in fathers, the mean age was 33.71 (SD = 4.92) years old, ranging from 23 to 51 years old. The annual average household income was 31880.00 RMB.

**Table 1:** Demographic characteristics of children with ASD and their families

		*n*	%
Gender	Male	248	85.5
	Female	42	14.5
Age	1-3	93	32.1
	4-6	133	45.9
	7-9	50	17.2
	≥ 10	14	4.8
Race/ethnicity	Han Chinese	279	96.2
	Minority Chinese	11	3.8
Number of children	Single child	255	87.9
	Non-single child	35	12.1
Family structure	Nuclear family	208	71.7
	Non-nuclear family	82	28.3
Address	Harbin	99	34.1
	Other cities	191	65.9
Urban/Rural areas	Urban	209	72.1
	Rural	81	27.9
Mother's education level	Elementary school	27	9.3
	Junior high school	97	33.4
	High school or technical school	68	23.4
	Vocational college or bachelor's degree	94	32.4
	Masters degree	4	1.4
Father's education level	Elementary school	17	5.9
	Junior high school	106	36.6
	High school or technical school	75	25.8
	Vocational college or bachelor's degree	85	29.3
	Masters degree	7	2.4

*Nuclear families consisted of a father, mother, and their children; non-nuclear families included families residing with grandparents.

Table 2 shows the annual per-capita household income and expenses for families with ASD children involved in the study. The per-capita household income of families involved in this study was 12,226.83 RMB and 4628 RMB, respectively, for families in urban and rural areas, whereas the values for the whole province were 10,245.28 RMB and 4132.29 RMB, respectively. Therefore, compared with the 2007 provincial statistics, 40.2% of the families from urban areas and 44.4% of the families from rural areas reported a higher annual per-capita household income.

**Table 2:** Per-capita household income and expenditure for participating families and comparison with provincial statistics for 2007

	Urban areas (RMB)	Rural areas (RMB)
Per-capita income		
Per-capita household income	12,226.83	4628.39
Provincial statistics for 2007	10,245.28	4132.29
Per-capita expense		
Per-capita household expense	10,919.23	7317.41
Provincial statistics for 2007	7519.28	3117.44

Among the families involved in this study, the per-capita household expense was 10,919.23 RMB for those in urban areas and 7317.41 RMB for those in rural areas. Meanwhile, the 2007 provincial statistics showed that the per-capita expenditures of residents in urban and rural areas were 7519.28 RMB and 3117.44 RMB, respectively. Therefore, 60.8% of the families from urban areas and 74.7% of those from rural areas involved in our report described higher per-capita household expenditures than in the overall provincial statistics.

**Figure 1:**
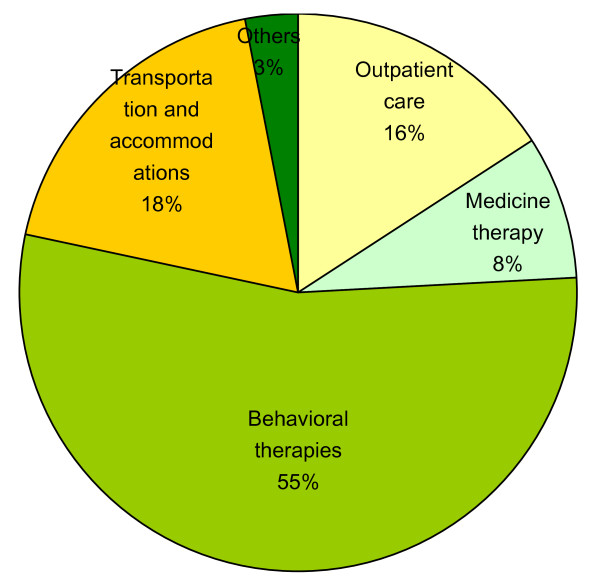
**Percentages of health care expenditure for various ASD related services**.

Overall, 60.2% of the participating families reported having spent more than 10,000 RMB on health services related to ASD in the last 12 months. As shown in Figure 1 and Table 3, the mean annual health service expenditure for children with ASD was 17,292.67 RMB. Of this, 11,072.37 RMB resulted from behavioral therapy, which accounted for 54.3% of all health care-related costs for ASD children. Other costs included outpatient care (1532.44 RMB), prescription medications (1240.65 RMB), and transportation and accommodation (2770.62 RMB). It is particularly interesting that no significant difference was found between urban and rural families in terms of either total household expenditure or health care-related expenditure (Table 3).

**Table 3:** Annual family expenditure on ASD related health services, overall and by residing areas

	*n*	Total	Outpatient care	Medicine	Behavioral therapies	Transportation and accommodation
Urban areas	178	17,497.28^†^	1637.53	1420.22	11,089.91	2701.08
Rural areas	68	16,757.06	1257.35	770.59	11,026.47	2952.65
Total	246	17,292.67	1532.44	1240.65	11,072.37	2770.62

Values are means. *Data were missing for 44 families; ^†^*P *> 0.05

As shown in Table 4, the mean percentage of health costs relative to annual family income was 70.5% for families from urban areas, and 128.5% for families from rural areas. We also found that the annual health care costs associated with ASD exceeded the annual family income in 19.9% of urban families and 38.2% of rural families, with 6.8% of urban families and 23.5% of rural families having costs that were two times higher than their annual family income. Among the participating families, the mean percentage of the families' health care expenditure for ASD relative to annual family expenditure was 44.1% in urban areas and 51.1% in rural areas. Comparison with provincial statistics reveals that 89.3% of the families reported a larger share of health care-related costs in overall household expenditures in urban areas and 88.1% in rural areas.

**Table 4:** Percentages **of **Health care expenditure in household income and total household expenditure*

	Mean (%)	Thresholds (%)	*N *(%)
Per-capita health expenditure as a percentage of per-capita household income			
Urban	70.5	< 50	94 (53.4)
		50-100	47 (26.7)
		100-150	19 (10.8)
		150-200	4 (2.3)
		≥ 200	12 (6.8)
Rural	128.5	< 50	21 (30.9)
		50-100	21 (30.9)
		100-150	3 (4.4)
		150-200	7 (10.3)
		≥ 200	16 (23.5)
Per-capita health care expenditure as a percentage of per-capita household expenses (2007 provincial statistics)^†^			
Urban	44.1	< 9.7	19 (10.7)
		≥ 9.7	158 (89.3)
Rural	51.1	< 8.7	8 (11.9)
		≥ 8.7	59 (88.1)

*Data were missing for 46 families; ^†^Compared with provincial statistics for 2007, the per-capita health expenses accounted for 9.7% of all household expenses in urban areas and 8.7% in rural areas.

## 4. Discussion

Although several studies have examined the prevalence of ASD, very little is known about the costs associated with these disorders or the economic burden on families with ASD children, particularly in China. Results of this study show a considerable economic burden of ASD. All participating families paid for all health care expenses; behavioral therapy accounted for the largest share of these costs. Of paramount concern is that the health care costs related to ASD actually exceeded annual per-capita household income in 19.9 percent of urban families and 38.2 percent of rural families. In fact, majority of families (89.3% of urban and 88.1% of rural areas) reported a higher proportion of ASD-related costs relative to overall household expenditure compared with provincial statistics.

The prevalence of ASD has increased from 4 to 5 per 10000 in 1960s to approximately 1-2% today [14-20]. It was estimated that there were about 67,000,000 ASD cases in the world (http://www.autismspeaks.org/). In the developed countries, ASD is publicly funded; most of the health related expenses are covered by the governments. In China, presently health services for children with ASD are not included in the national health insurance program. Therefore, administrative data related to health service use and expenses are unavailable for ASD children at the national level. Gregory et al. reported that the annual cost of autism for children in the US was $6132, including $3992 for outpatient care, $869 for physician visits, and $971 for prescription medications. Notably, the expenses for children with ASD were higher than those for children without ASD [21]. It was also reported that US families with autistic children spent $613 on out-of-pocket expenses each year, corresponding to 9.0% of total expenditure [22]. This is much lower than the shares of income observed in the present study. Some reports have described that children with autism incurred 2.5 times higher outpatient costs, 2.9 times higher inpatient costs, and 7.6 times higher medication costs than randomly selected children without autism [23]. In terms of special health services, families with autistic children spent more than families in general [24,25].

Results of the present study show that costs related to behavioral therapies constituted the greatest share of overall ASD related health care costs (54.3%) followed by transportation and accommodation (18.4%), outpatient care (15.9%), and medication (8.2%). Similarly, Ganz et al. reported that behavioral therapies comprised the largest component of direct medical costs in their study conducted in the USA [12]. Wang reported that although the per patient expenditures grew slightly over time, the increased prevalence of ASD may have contributed to the staggering rise in total ASD-associated health care expenditures [26]. We consider that the high share of behavioral therapy costs is reasonable because, as described previously, parents now believe that earlier diagnosis and rehabilitation are particularly important for children with ASD [27] and that behavioral therapies are the key to rehabilitation, especially in children aged 3-6 years [28].

Transportation and accommodation (18.41%) comprised a greater proportion of total health care costs than outpatient costs and consulting (15.94%). This is perhaps not surprising because many of the families did not live in Harbin, where the CDBRC is located. For that reason, they incurred high transportation and accommodation costs. The CDPF has planned to pilot the model of ASD rehabilitation centers in 31 major cities in China and to expand it to other cities if it is successful. The goal of the plan was to ensure that the families with ASD children could seek help and receive services in the areas where they live. As part of it, transportation and other related costs could be reduced, especially for those who live in rural areas.

In developed countries, the costs of ASD services are covered by the governments. In China, the families of ASD children pay the costs of ASD rehabilitation out of their own pockets, which account for a significant proportion of their household income. Since 2006, the CDPF has initiated rehabilitation support programs for ASD for low income families. As part of the program, each ASD child may receive about 10,000 RMB each year to cover the rehabilitation costs. Additionally, the Harbin municipal government has provided 8000 RMB /year to each ASD child on top of the national support since 2010.

Montes reported the average loss of annual income associated with having a child with autism spectrum disorder was $6200 or 14% of their household income [29]. Unfortunately, we cannot compare the health care expenses incurred by the families in this study directly with those of studies conducted in developed countries because of different economic conditions and health insurance policies. Nevertheless, we can evaluate economic burdens based on the health care expenditures as a percentage of family income and expenditure. The per-capita household income of our families was equivalent to the mean provincial income. However, the health care costs for ASD children in some families were greater than their annual income, with costs exceeding twice of the household income in some families. Moreover, health expenditure as a share of total family expenses was much higher for families with ASD children than the provincial level and exceeded the mean proportion for Heilongjiang province in > 80% of families, irrespective of whether they lived in urban or rural areas. Consequently, the families were compelled to reduce their other expenses.

Although no difference was found in health care expenditure between urban and rural families, families from rural areas reported lower income than those from urban areas. Moreover, transportation and accommodation costs were higher for families from rural areas than for those from urban areas. Therefore, the percentage of ASD health care expenditures in total household income for rural families was 1.82 times higher than that for urban families. Consequently, rural families face a greater economic burden than urban families. In fact, many participating families reported using personal savings or private loans to cover the expenses.

This study was consistent with previous studies investigating economic burdens on families with ASD children. Studies in the USA showed that families with ASD children were in desperate need of financial support to gain access to treatment for their ASD children [23,30]. This burden was often exacerbated because many mothers with ASD children or foster parents were unable to work outside the home as a result of the extensive need for care of their children. Future longitudinal studies are needed to investigate the changes in ASD disease burden and how the changes are related to the prognosis of ASD.

### 4.1. Study limitations

Some limitations of the study warrant comment. First, this is a single center study. Participants were those who attended the CDBRC. ASD children who received services from private practitioners were not included in our study. Therefore, generalization of the results is limited and caution must be taken when comparing them with results obtained for other regions. However, data about health related costs include not only expenses incurred at the CDBRC, but also the costs related to the services received at other organizations. Second, the data relied on self-reporting, which directly implies that recall and reporting biases are possible. Participants may not be able to remember exactly specific cost items. However, the overall expenses reported by the participants may not deviate significantly from the true expenses. Third, no comparison group consisting of children with other chronic conditions was used for this study. Therefore, we cannot determine whether ASD is associated with greater or lesser costs than other health conditions. Such studies should be included in future research efforts.

## 5. Conclusion

This study is the first of this kind in China to examine the economic burden of ASD on families. Our findings shed some light on the circumstances faced by families with ASD children. With the rapidly increasing demand for health services for ASD children, the economic burden imposed on their families will become an important social issue. Therefore, future studies should also consider examining family stress, quality of life, and lost income for caregivers in this population. Health services for ASD children should be considered in the future development of medical insurance coverage in China. Currently, the families use their personal savings and loans to cover the costs because health care expenditures exceed the household income in many families. The costs associated with treatment affect their socioeconomic status, causing them to fall below the poverty level, leading to a vicious circle. We believe that more studies are needed in China to understand the impact of ASD on the patients and their families better, and thereby to aid the development of health and insurance policies.

## Competing interests

The authors declare that they have no competing interests.

## Authors' contributions

Study concept and design: LJW. Acquisition of data: JW. Analysis and interpretation of data: JW. First draft of manuscript: JW and XZ. Critical revision of the manuscript for important intellectual content and interpretation: LJW, JLW and AT. Statistical analysis: WX and CHS. Administrative, technical, and material support: LJW. Study supervision: LJ W.

All authors read and approved the final manuscript.

## Pre-publication history

The pre-publication history for this paper can be accessed here:

http://www.biomedcentral.com/1472-6963/12/7/prepub
